# A Real-Time PCR Assay for the Detection of Atypical Strains of *Chlamydiaceae* from Pigeons

**DOI:** 10.1371/journal.pone.0058741

**Published:** 2013-03-14

**Authors:** Aleksandar Zocevic, Fabien Vorimore, Nadia Vicari, Julien Gasparini, Lisa Jacquin, Konrad Sachse, Simone Magnino, Karine Laroucau

**Affiliations:** 1 University Paris-Est, Anses, Animal Health Laboratory, Bacterial Zoonoses Unit, Maisons-Alfort, France; 2 Istituto Zooprofilattico Sperimentale della Lombardia e dell'Emilia Romagna “Bruno Ubertini”, Pavia, Italy; 3 Laboratoire Ecologie & Evolution, CNRS ENS UPMC UMR 7625, Paris, France; 4 Friedrich-Loeffler-Institut (Federal Research Institute for Animal Health), Institute of Molecular Pathogenesis, Jena, Germany; Washington State University, United States of America

## Abstract

Recent evidence of the occurrence of atypical *Chlamydiaceae* strains in pigeons, different from the established *Chlamydiaceae*, requires the development of a specific and rapid detection tool to investigate their prevalence and significance. Here is described a new real-time PCR assay that allows specific detection of atypical *Chlamydiaceae* from pigeons. The assay has been used to assess the dissemination of these strains in field samples collected from Parisian pigeon populations in 2009. The results suggest a limited dissemination compared to the usually higher prevalence of *Chlamydia psittaci* that is the main species associated with avian chlamydiosis.

## Introduction

Avian chlamydiosis is a zoonotic disease caused by *Chlamydia psittaci*, an obligatory intracellular bacterium and member of the family *Chlamydiaceae* [1]. The infection is usually systemic and occasionally fatal in birds. The clinical signs vary greatly in severity and depend on the species and age of the birds as well as the causative strain [2]. Transmission of *C. psittaci* from birds to humans is reported regularly, particularly in high-risk individuals such as breeders or slaughterhouse workers [3]. Although the importance of *C. psittaci* as the causative agent of avian chlamydiosis in birds has been known for decades [4], several studies have recently provided evidence of the occurrence of other chlamydial species in birds such as *C. abortus* [5], [6], *C. suis* and *C. muridarum* [7] as well as *C. pecorum* and *C. trachomatis* [8]. Additionally, atypical strains of *Chlamydiaceae* were recently detected in chickens [9], [10], [11] and pigeons [8], [12], [13], [14].

A better characterisation of these atypical *Chlamydiaceae* and the availability of fast, sensitive and specific detection tools are needed in order to better understand their epidemiological importance. A specific 16S rDNA-based real-time PCR was developed recently for the specific detection of atypical *Chlamydiaceae* from chickens (hereafter named ACC) [14].

The work reported in this paper deals with the development and validation for routine diagnostic purposes of a real-time PCR based on the enolase A (*eno*A) gene target for the specific detection of atypical *Chlamydiaceae* from pigeons (hereafter named ACP). This assay has been used to investigate the dissemination of this agent in Parisian pigeon populations.

## Methods

### 1. Microorganisms

Chlamydial strains and isolates used for determination of specificity, sensitivity and detection limit of the new ACP-specific real-time PCR assay are listed in **Tables 1** **and** **2**. *Chlamydiaceae* were propagated in the yolk sac of 7 day-old embryonated chicken eggs and stored at −80°C. Non-chlamydial bacterial and fungal strains obtained from the University of Tours (France) and the Anses strain collection (Maisons-Alfort, France) were used for specificity testing. These included: *Escherichia coli*, *Proteus mirabilis*, *Proteus vulgaris*, *Klebsiella pneumoniae*, *Salmonella enteritidis*, *Salmonella enteritidis serovar Gallinarum*, *Salmonella enteritidis serovar Typhi*, *Serratia marcescens*, *Morganella morganii*, *Citrobacter freundii*, *Yersinia enterocolitica*, *Yersinia pseudotuberculosis*, *Acinetobacter baumannii*, *Pasteurella haemolytica*, *Bordetella bronchiseptica*, *Staphylococcus aureus*, *Streptococcus pyogenes*, *Enterococcus faecalis*, *Mycobacterium avium subsp. avium*, *Aspergillus fumigatus*, *Aspergillus niger* and *Candida albicans*.

**Table 1:** Characteristics of ACP isolates included in this study.

ID	Country	Year of Isolation	Initial sample
10–743/SC13	France	2010	Cloacal swab
10–881/SC42	France	2010	Cloacal swab
DC96	Germany	2011	Spleen
DC97	Germany	2012	Faeces
PV_2806/51	Italy	1996	Intestinal content
PV_2863/2	Italy	1996	Intestinal content
PV_3515/3	Italy	1996	Intestinal content
PV_3954/22	Italy	1996	Intestinal content
PV_7341/13	Italy	1997	Intestine
PV_7344/2	Italy	1997	Intestine
PV_155757/2010	Italy	2010	Pool of organs
PV_48558/2010	Italy	2010	Pool of organs
PV_58394/2012	Italy	2012	Pool of organs

**Table 2:** *Chlamydial* strains and isolates used for determination of specificity of the ACP-specific real-time PCR assay.

Species	Strains/isolates
Atypical *Chlamydia* from chicken (ACC)	08–1274/3^a^, 08–1274/13^a^, 08–1274/19^a^, 08–1274/21^a^, 08–1274/22^a^, 08-1274/23^a^
*C. psittaci*	Loth^b^, VS1^c^, CP3^c^, GR9^c^, TT3^c^, NJ1^c^, Cal 10^c^, VS225^c^, 06–889^a^
*C. abortus*	AB7^c^, 1B^c^, LLG^d^, S26/3^c^
*C. pecorum*	824^c^, AB10^c^, iB3^c^, iB3^c^, LW679^c^, SBE^c^
*C. felis*	Dohycat^e^
*C. caviae*	GPIC^c^, 98–3196/1^a^

Sources: **^a^** Anses Maisons-Alfort, France; ^b^ CHU Amiens, France; ^c^ INRA Tours, France; ^d^ Faculty of Veterinary Medicine – Aristotle University of Thessaloniki, Greece; ^e^ Vaccine strain.

### 2. Preparation of genomic DNA

Chlamydial strains cultivated onto vitellus membranes as well as biological samples were subjected to DNA extraction using the QIAamp DNA Mini Kit (Qiagen, Courteboeuf, France). DNA extracts were stored at −20°C before analysis.

DNA of non-chlamydial samples was extracted using the Instagen Matrix DNA Kit (BioRad, Marnes-la-Coquette, France) according to the manufacturer's instructions. DNA concentration and purity of non-chlamydial extracts were spectrophotometrically assessed by reading A260 and A280 and confirmed by visualization on 0.5% (w/v) agarose gels. Finally, DNAs with a mean concentration of 400 µg/ml were stored at −20°C until required for analysis.

### 3. Sequencing of the *eno*A gene locus

YP_enoA5 and YP_enoA6 used for amplification of an approximately 400-bp segment of the *eno*A gene were previously published by Pannekoek and colleagues [15]. PCR reactions were performed in a final volume of 25 µl. The mix contained 2 µl of DNA template, 2.5 µl of 10× PCR reaction buffer, 1.25 U of HotStarTaq DNA Polymerase (Qiagen, France), 100 µM of each deoxynucleotide triphosphate and 0.5 µM of each primer. The conditions used for amplification were 95°C for 1 min, 45 cycles of 94°C for 30 s, 48°C for 1 min, 72°C for 1 min and 30 s, 72°C for 10 min (final extension). PCR-amplified segments of *eno*A were sequenced by Eurofins MWG Operon (Nantes, France). Sequence alignments were conducted using the Bionumerics software package version 4.6 (Applied-Maths, Belgium). A dendrogram including one representative of each *eno*A sequence type for established *Chlamydiaceae* strains [15], [16], one representative for ACC [14] and one for ACP was constructed using the Unweighted Pair Group Method with Arithmetic Mean algorithm. Nucleotide sequences have been deposited under GenBank accession numbers HE983370 to HE983383.

### 4. ACP-specific real-time PCR

Specific primers ACP_Fw (5‘-CATGCAAGCTATTGAGAAAAGTGGT -3’) and ACP_Rv (5′- CCTTGATATGTACGTGTTTTCTCG -3′) as well as a specific probe ACP_Pr (5′-FAM- CACCCCTGGTGAAGATATTTCCTTAGCAT -TAMRA 3′) were designed based on *eno*A and synthesised by Eurofins MWG Operon. Amplification was performed using the 2× TaqMan Universal PCR Master Mix (Applied Biosystems, Courtaboeuf, France). The final volume of the reaction mixture amounted to 20 µl including 10 µl of master mix, 2 µl of DNA sample, 0.6 µM of each primer, 0.1 µM of the probe and sterile PCR water added to reach the final volume. Amplification was carried out at an ABI Prism 7000 thermocycler (Applied Biosystems) using the following cycling parameters: heating at 95 °C for 10 min, 50 cycles of 95°C for 15 s and 60°C for 1 min. For all PCR reactions, DNA of the isolate 10–743/SC13 arbitrarily defined as reference strain for ACP was used as a positive control while sterile water was used as negative control.

### 5. Performance evaluation of the ACP-specific real-time PCR

The detection limit of the real-time PCR was determined in triplicate (independent runs) using decimal serial dilutions of purified genomic DNA from 10–743/SC13 suspensions equivalent to 3×10^−1^ to 3×10^5^ inclusion-forming units (IFUs) per PCR reaction. The concentration of this DNA was previously determined by comparison to a DNA purified from cell culture containing defined numbers of IFUs of *C. psittaci*. The standard curve and the number of IFUs were automatically determined by the software using manually entered IFU standard concentrations. The standard curve was generated by plotting Ct against the logarithm of IFU per PCR reaction mix. The Ct, at which the fluorescence crosses the threshold value, and the lower limit of detection of the assay were calculated automatically.

The specificity of the new real-time PCR was evaluated on 22 *Chlamydiaceae* DNA samples (**Table 2**) yielding Ct values between 22 and 30 when tested with the 23S rRNA-based *Chlamydiaceae*-specific real-time PCR (*Chlamydiaceae* 23S-rtPCR) [17], and 22 non-chlamydial microorganisms.

DNA of strain 10–743/SC13 was amplified 30 times in the same run to determine intra-assay repeatability, and 11 times in separate runs on different days for inter-assay reproducibility. The respective CVs were then calculated.

### 6. *Chlamydiaceae* field samples

A panel of 125 DNAs extracted from cloacal swab samples collected from pigeons in a previous study [13] was used to evaluate the dissemination of the ACP. These samples, which contained an internal positive control (Exogenous internal positive control, TaqMan, Life technologies), were previously tested positive in the *Chlamydiaceae* 23S-rtPCR [17].

## Results and Discussion

**Figure 1:**
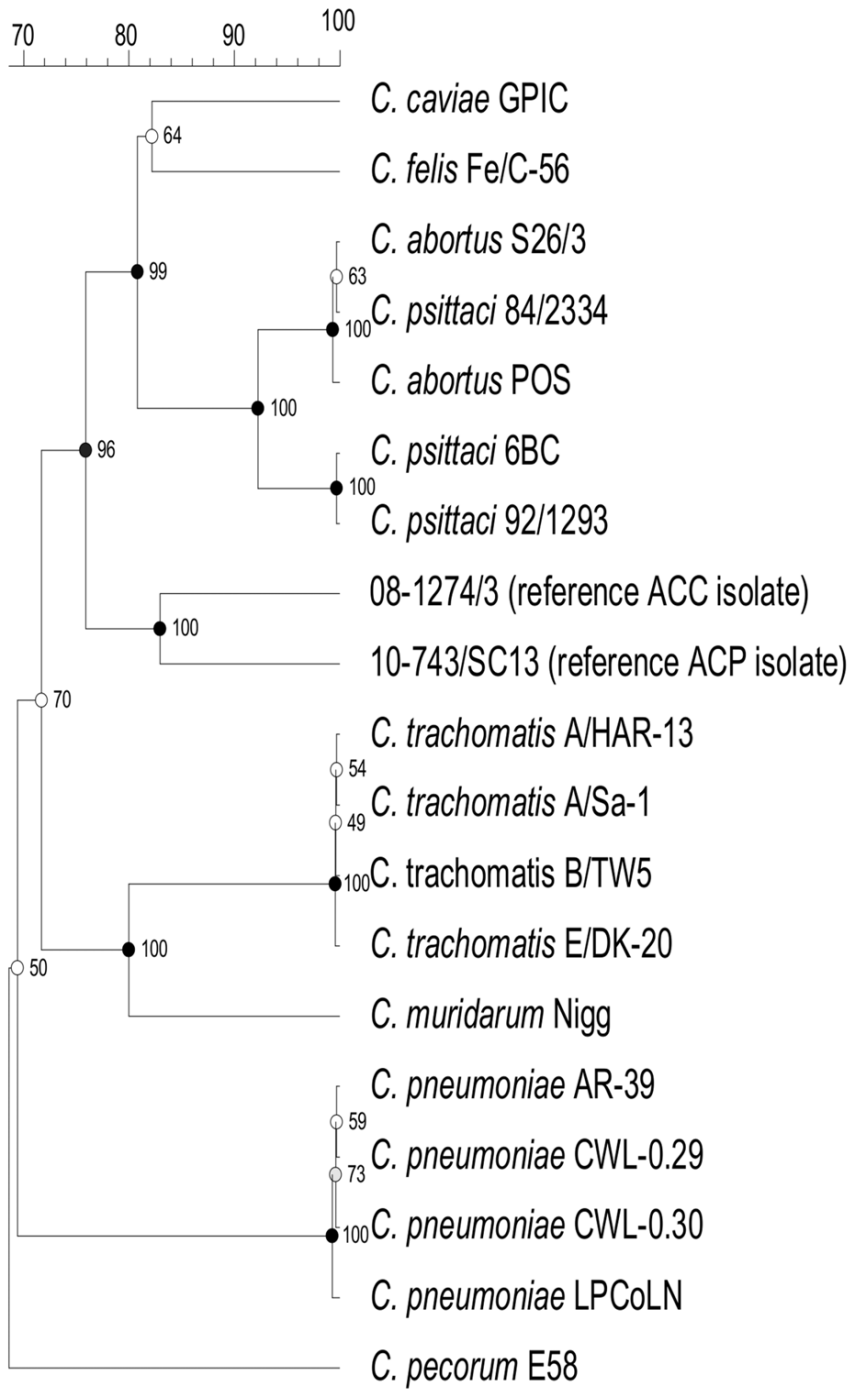
Phylogenetic analysis of the *eno*A sequence for ACC (08–1274/3 reference), ACP (10–743/SC13 reference) and various established *Chlamydiaceae* strains (one representative of each *eno*A sequence type; [**15]**, [16]). Bootstrap test was for 1000 repetitions. Horizontal distances correspond to genetic distances expressed in percentage of sequence similarity, vertical distances are arbitrary.

Recent evidence of the occurrence of atypical *Chlamydiaceae* strains in chickens and pigeons highlighted the necessity of specific and fast detection tools for the investigation of their epidemiological and clinical importance. Thirteen ACP strains have been isolated up to now in France, Germany and Italy (**Table 1**). A similar approach to the one used for ACC [14] has been adopted in this study, except that the enolase A (*eno*A) gene has been selected as target. This gene locus had been previously included in a Multi-Locus Sequence Typing scheme for *Chlamydia* spp. [15], [16]. All *eno*A sequences of ACP strains in **Table 1** proved identical. The consensus sequence was compared *in silico* to those of the nine established *Chlamydiaceae* species [15], [16] and also to ACC sequences [14]. Alignment (**Figure S1**) and phylogenetic analysis (**Figure 1**) revealed that this sequence was specific to ACP and could be a convenient target for their unambiguous identification by real-time PCR.

The specificity of the newly developed *eno*A-based real-time PCR assay (*eno*A-rtPCR) was tested with genomic DNA extracts of several bacterial and fungal species. Neither the DNA samples from non-chlamydial species (n = 22), nor the DNA samples from *C. psittaci* (n = 9), *C. abortus* (n = 4), *C. pecorum* (n = 6), *C. felis* (n = 1), *C. caviae* (n = 2) and newly described atypical chicken (ACC) isolates included in this study (n = 6) gave rise to a measurable signal. As expected, all DNA extracts from French (n = 2), German (n = 2) and Italian (n = 9) ACP isolates (**Table 1**) were successfully amplified (data not shown).

**Figure 2:**
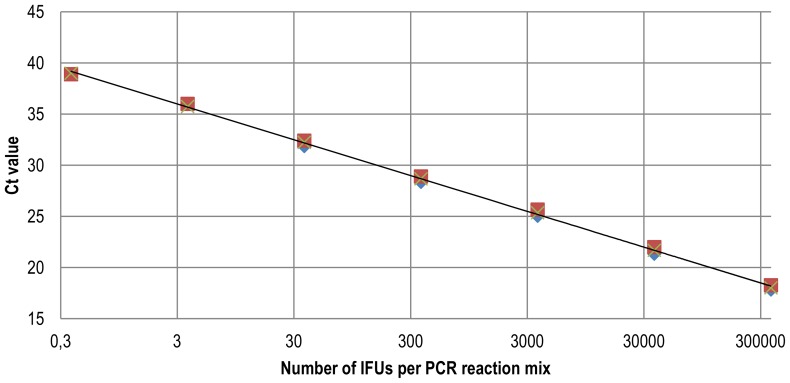
Real-time PCR sensitivity testing to detect genomic DNA of ACP isolate 10–743/SC13. DNA was extracted and serially diluted. The concentration (IFU) was calculated from the Ct value based on the calibration curve from serial 10-fold dilutions of a DNA purified from cell culture containing defined numbers of IFUs of *C. psittaci*. Each dilution was subjected to real-time PCR analysis in triplicate. The r^2^ linearity value from the linear regression is 0.9987. y = −3.5111×log (x) +37,702, efficiency  = 10(−1/slope)−1 = 93%.

In order to determine the detection limit of the new real-time PCR, a decimal dilution series of the titrated 10–743/SC13 DNA was tested. The results showed that the assay was able to detect the DNA from 3 IFUs of the respective pathogen per PCR reaction with an efficiency of 93% (**Figure 2**). The intra-assay and inter-assay CVs were 2.5% and 1.9% respectively. Therefore, the real-time PCR assay presented here has proved to be sensitive, specific and reproducible and offers the possibility of assessing the epidemiological importance of ACP infections in pigeon populations.

To evaluate the suitability of the assay for this purpose, a retrospective examination of previously collected field samples in Parisian feral pigeon populations [13] has been carried out. Out of the 125 *Chlamydiaceae*-positive samples, 10 were positive (8%) with the new ACP-specific real-time PCR (**Table 3**), including samples 09–489/LP23 and 09–589/S46 that had previously been identified as ACP by partial *omp*A and 16S rRNA sequencing [13]. These findings suggest a low dissemination of ACP in comparison with *C. psittaci,* which prevalence of 68% had been determined (**Table 3**) using the *inc*A-based *C. psittaci-*specific real-time PCR (*inc*A-rtPCR *C.psittaci*) according to Menard and colleagues [18]. A mixed infection involving an ACP strain and *C. psittaci* was also found in two samples from Genevilliers (**Table 3**).

**Table 3:** Summary of results from the study on urban pigeons from Paris.

City (district number)	23S-rtPCR *Chlamydiaceae*	*inc*A-rtPCR *C.psittaci*	*eno*A-rtPCR ACP
	No. of +ve samples*	No. of +ve samples*	No. of +ve samples
Clamart_La Plaine (92)	10/29	8/10	1/10
Clamart_Maison Blanche (92)	9/41	6/9	1/9
Clamart_Trivaux (92)	5/25	5/5	0/5
Courbevoie (92)	15/60	11/15	1/15
Creil (60)	16/66	13/16	1/16
Fontenay sous bois_La Fontaine (93)	7/77	6/7	0/7
Genevilliers (92)	23/142	12/23	6/12
Pantin (93)	4/71	4/4	0/4
Paris_Jussieu (V)	13/69	7/13	0/13
Paris_La Roquette (XI)	3/11	1/3	0/3
Paris_Montreuil (XX)	9/21	4/9	0/9
Paris_Sacré Cœur (XVIII)	5/19	3/5	0/5
Paris_St Denis (X)	2/36	1/2	0/2
Paris_Vanves (XIV)	4/27	4/4	0/4
**Total**	**125/694** (18%)	**85/125** (68%)	**10/125** (8%)

* see reference [13]

Given the low number of samples tested and the limited geographical range covered, the results of the present study can only be regarded as preliminary. Atypical pigeon strains have been isolated in the laboratories of the authors from the intestinal content or organs of necropsied urban pigeons. Their pathogenicity for pigeons is unknown. Another open issue is the host range. A recently isolated atypical parrot strain from Germany (designated 10DC88) was found to possess an *eno*A sequence identical to those of ACP, so that it was positive with the ACP-specific real-time PCR assay. This indicates that ACP seem not to be restricted to pigeons and could possibly be circulating in other avian hosts.

All in all, as a rapid and specific diagnostic tool is now available, it seems straightforward to include ACP investigation to analyses of diagnostic samples from birds and to undertake systematic studies to characterize the epidemiology and etiology of ACP infections in avian populations.

## Conclusions

A sensitive and specific real-time PCR assay for the detection of atypical *Chlamydiaceae* from pigeons has been developed and preliminary validated. The use of this new tool could contribute to a better understanding of the importance of these bacterial agents.

## Supporting Information

Figure S1 **Alignment of partial** ***eno*** **A sequence for ACC (08–1274/3 reference), ACP (10–743/SC13 reference) and various established** ***Chlamydiaceae*** **strains (one representative of each enoA sequence type;** [**15]**, [16] **).** Nucleotide homologies are represented by dots. The numbers refer to alignment positions. ACP-specific primer sequences are boxed and the probe sequence is underlined.
(TIF)
