# Supplementary figures and images for: A Real-Time PCR Assay for the Detection of Atypical Strains of Chlamydiaceae from Pigeons
Source: PLoS One. 2013 Mar 14;8(3):e58741. doi: 10.1371/journal.pone.0058741 (PMC3597529; doi:10.1371/journal.pone.0058741)

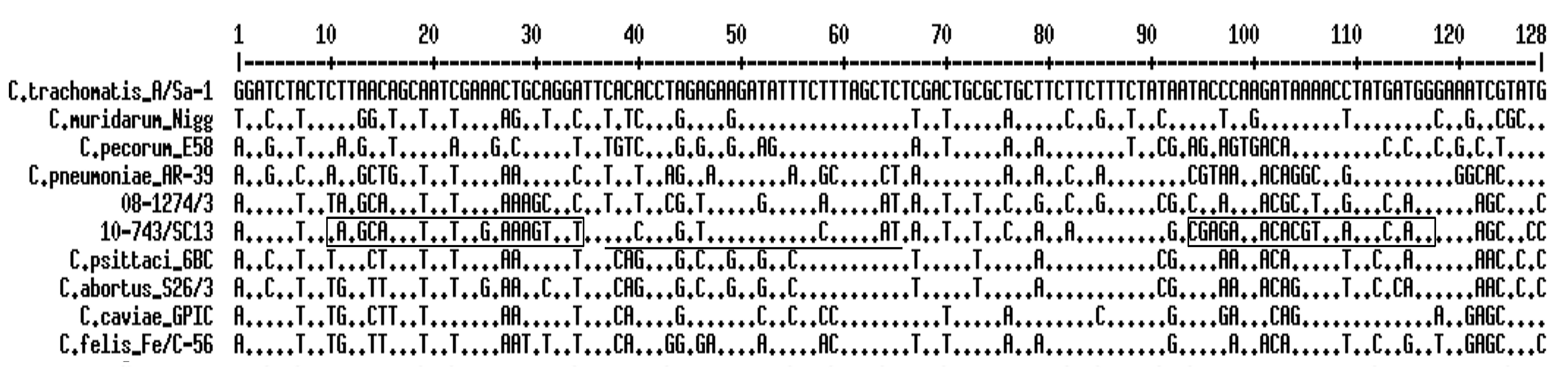

Supplement: Figure S1 — Alignment of partial eno A sequence for ACC (08–1274/3 reference), ACP (10–743/SC13 reference) and various established Chlamydiaceae strains (one representative of each enoA sequence type; [15] , [16] ). Nucleotide homologies are represented by dots. The numbers refer to alignment positions. ACP-specific primer sequences are boxed and the probe sequence is underlined. (TIF) [file pone.0058741.s001.tif]
